# Mortality of Cities

**Published:** 1850-09

**Authors:** 


*Mortality of Cities.*—It is stated by a writer in a New York paper that the weekly mortality of that city is 1 to every 1,289 inhabitants, while in London it is only 1 to every 2,600. Upon which the New York Medical Gazette makes the following remarks:

"There are two or three causes which go to produce the disproportion noticed above. 1st. The number of immigrants (from one to two hundred thousand annually) landed among us, many of them in a debilitated state, and liable to sicken from exposure and change of climate. 2d. The difference in the habits of the Londoners and New Yorkers, the former taking life more easily and comfortably by fai than our steam-engine citizens. 3d. The variable character of our climate, as contrasted with the moist equable atmosphere of London. This, however, is of less importance than the two previous reasons. We do not see that New York need be more unhealthy than London, when the tide of immigration shall have slackened, and our people have carried the art of living to greater perfection. It would be an interesting question to ascertain the relative proportion of physicians and inhabitants, in this City and London.

